# Expression of Heat Shock Protein 27 in Melanoma Metastases Is Associated with Overall Response to Bevacizumab Monotherapy: Analyses of Predictive Markers in a Clinical Phase II Study

**DOI:** 10.1371/journal.pone.0155242

**Published:** 2016-05-11

**Authors:** Cornelia Schuster, Lars A. Akslen, Oddbjørn Straume

**Affiliations:** 1 Centre for Cancer Biomarkers CCBIO, Department of Clinical Medicine, University of Bergen, Bergen, Norway; 2 Department of Pathology, Haukeland University Hospital, Bergen, Norway; 3 Department of Oncology and Medical Physics, Haukeland University Hospital, Bergen, Norway; 4 Centre for Cancer Biomarkers CCBIO, Department of Clinical Science, University of Bergen, Bergen, Norway; University of Queensland Diamantina Institute, AUSTRALIA

## Abstract

The aim of this study was to identify potential predictive biomarkers in 35 patients with metastatic melanoma treated with anti-angiogenic bevacizumab monotherapy in a clinical phase II study. The immunohistochemical expression of various angiogenic factors in tissues from primary melanomas and metastases as well as their concentration in blood samples were examined. Strong expression of Heat Shock Protein 27 (HSP27) in metastases correlated significantly with complete or partial response to bevacizumab (p = 0.044). Furthermore, clinical benefit, i.e., complete or partial response or stable disease for at least 6 months, was more frequent in patients with strong expression of HSP27 in primary tumors (p = 0.046). Tissue expression of vascular endothelial growth factor (VEGF-A), its splicing variant VEGF165b or basic fibroblast growth factor (bFGF) did not correlate with response, and the concentration of HSP27, VEGF-A or bFGF measured in blood samples before treatment did not show predictive value. Further, microvessel density, proliferating microvessel density and presence of glomeruloid microvascular proliferations were assessed in sections of primary tumors and metastases. Microvessel density in primary melanomas was significantly higher in patients with clinical benefit than in non-responders (p = 0.042). In conclusion, our findings suggest that strong HSP27 expression in melanoma metastases predicts response to bevacizumab treatment.

**Trial Registration:**

ClinicalTrials.gov NCT00139360

## Introduction

Cutaneous malignant melanoma (CMM) has shown an increasing incidence worldwide among fair skinned populations during the last decades [1]. Five-year survival in metastatic disease is still poor [2], and it remains to be seen whether new options like targeted treatment [3] or immune checkpoint blockade [4] will improve long term survival rates. Acquired resistance [5] and initial low response rates [6] are still major reasons for poor outcome, and predictive biomarkers in addition to *BRAF* mutation status are needed [3].

Angiogenesis is an important cancer hallmark and treatment target [7, 8]. Preclinical models and clinical investigations have characterized primary melanomas and metastases as highly vascularized [9–11]. Since vascular endothelial growth factor A (VEGF-A) plays a key role in angiogenesis [12, 13] and is expressed in a high proportion of melanomas [9], we conducted a clinical trial with bevacizumab monotherapy, a humanized monoclonal antibody that binds specifically to VEGF-A, in patients with metastatic CMM [14]. As published previously, we observed a clinical benefit rate of 31% [14], indicating that VEGF-A driven angiogenesis is important in a subgroup of these patients. In addition, efficacy of different combinations between bevacizumab and chemotherapy in patients with metastatic melanoma has been reported [15–18]. Bevacizumab is also implemented in the treatment of various other solid tumors but still no predictive biomarkers have been validated [19, 20].

In the present study, we aimed to explore potential predictive biomarkers known to be involved in angiogenesis, and we focused on VEGF-A [12, 13], its splicing variant VEGF165b that binds competitively to VEGFR-2 without phosphorylating pro-angiogenic pathways [21], basic fibroblast growth factor (bFGF) [8] and Heat Shock Protein 27 (HSP27).

HSP27, a small heat shock protein, maintains cell survival under stressful conditions by management of misfolded proteins and prevention of apoptosis [22, 23]. Furthermore, it appears to play an important role in angiogenesis and in tumor cell migration as well as in organization of the cytoskeleton [23, 24]. HSP27 expression is associated with impaired prognosis in melanoma and other tumors as well as resistance to chemotherapy [23, 24]. Previous studies from our group have identified HSP27 as important for tumor dormancy, angiogenesis regulation and tumor progress in cutaneous melanoma and breast cancer [24]. Downregulation of HSP27 in an angiogenic breast cancer cell line resulted in reduced secretion of VEGF-A and bFGF, supporting a HSP27 dependent co-regulation of these factors. Furthermore, the expression of HSP27-related transcription factors phospho-STAT3 and NFkB, involved in regulation of angiogenesis, were significantly reduced in xenograft tumors from HSP27 knockdown cells [24]. Others showed increased secretion of VEGF after endothelial cells were exposed to extracellular HSP27 [25].

Importantly, tissue based angiogenesis markers like microvessel density (MVD), proliferating microvessel density (pMVD), vascular proliferation index (VPI) and presence of glomeruloid microvascular proliferation (GMP) [11, 26–28] were studied. To our knowledge, the present study is the first to indicate that strong tissue expression of HSP27 in melanoma metastases predicts overall response to treatment with bevacizumab monotherapy. However, several other angiogenic markers were not predictive in our study.

## Material and Methods

### Patients and study design

Thirty-five patients with metastatic melanoma were enrolled in an open-label, single arm phase II study at Haukeland University Hospital, Norway, and were treated with bevacizumab 10 mg/kg q14d until disease progression or intolerable toxicity (ClinicalTrials.gov: NCT00139360). Study design, eligibility criteria and clinical response data were reported earlier [14]. Fourteen of the thirty-five patients were treated with dacarbazine before they were included in the study. One of these patients received bevacizumab as third line treatment. Twenty-one patients were treated with bevacizumab first line. This approach was based on the two-stage design for phase II clinical trials by Simon [29]. As published previously [14], six of the thirty-five patients had an overall response (OR), *i*.*e*. complete (CR) or partial response (PR), following treatment with bevacizumab monotherapy. In addition, five more patients had stable disease (SD) for at least six months. Thus, altogether 31% had a clinical benefit (CB), *i*.*e*. OR and SD. The study was conducted in accordance with the ethical principles of the Declaration of Helsinki and the International Conference on Harmonization of Good Clinical Practice, and approved by the Regional Ethics Committee (processing number: 05/329) and the Norwegian Medicines Agency. Informed consent was signed by all patients before enrolment.

### Tissue and blood samples

Paraffin embedded tissue of primary tumors was obtainable in 32 of 35 patients. The three cases missing primary tumor tissue blocks represent one primary ocular melanoma treated by radiation therapy, one unknown primary lesion, as well as one undiscoverable tissue block. All primary melanomas were reclassified (LAA, CS) and characterized by the following histopathologic features in hematoxylin and eosin-stained sections: histologic type by Clark, tumor thickness by Breslow, mitotic rate, ulceration, Clark’s level of invasion, growth phase, vascular invasion, tumor infiltrating lymphocytes and necrosis [30].

Tissue samples from metastases were available in all patients (35/35). The metastasis diagnosed closest to the date of inclusion (median 5 days) was chosen for further analysis if several metastatic lesions were available. The material includes core needle biopsies (n = 17), mostly taken from lung, liver and abdominal or pelvic organs, as well as excisional biopsies (n = 18) from skin metastases or lymph nodes.

In addition, plasma and serum samples were taken before the first treatment with bevacizumab (median: 2 days); altogether 29 plasma and 28 serum samples were available. EDTA blood was immediately centrifuged at +4°C for 10 minutes at 1600xg, serum samples were processed after clotting for 30 minutes at room temperature. All samples were stored at -20°C and aliquoted when used for analysis.

### Immunohistochemistry

Tissue sections of 4–5 μm were stained with primary antibodies for HSP27, VEGF-A, VEGF165b and bFGF. Furthermore, double staining with anti-factor VIII (F-VIII) and Ki67 was performed for angiogenesis assessment. After deparaffinizing in Xylene and different alcohol dilutions and rehydration, heat mediated or enzymatic antigen retrieval was performed. Endogenous peroxidase and alkaline phosphatase were blocked before incubation with the primary antibody followed by incubation with appropriate HRP-EnVision (DAKO, K4011 or K4007). For staining with HSP27 a secondary rabbit anti-goat antibody (Southern Biotech, Cat. no. 6164–01) was used; for double staining, a secondary goat anti-mouse antibody (Southern Biotech, Cat. no. 1031–04) was used. Details are provided in Table 1. For negative controls, primary antibodies were omitted or specific blocking peptides for HSP27, VEGF-A and bFGF were added. Tissues from different cancer types were used as positive controls.

**Table 1:** Immunohistochemical staining methods.

Antibody	Provider	Epitope retrieval	Dilution	Incubation
**HSP27 pAb sc-1048 (goat)**	**Santa Cruz**	**MW 6**^**th**^ **sense 20 min, pH6**	**1:100**	**30 min, RT**
**VEGF-A pAb sc-152 (rabbit)**	**Santa Cruz**	**MW 6**^**th**^ **sense 20 min, pH9**	**1:50**	**60 min, RT**
**VEGF165b mAb ab14994 (mouse)**	**Abcam**	**MW 6**^**th**^ **sense 20 min, pH9**	**1:100**	**Overnight, 4°C**
**FGF-2 pAb sc-1390 (rabbit)**	**Santa Cruz**	**Pressure cooker, pH9**	**1:50**	**60 min, RT**
**Von Willebrand Factor pAb A0082 (rabbit)**	**Dako**	**Proteinase K, 5 min**	**1:800**	**30 min, RT**
**Von Willebrand Factor pAb A0082 (rabbit) and Ki67 mAb (mouse) M7240**	**Dako**	**MW 6**^**th**^ **sense 20min, pH6**	**1:500 and 1:200**	**60 min, RT**

pAb, polyclonal antibody; MW, microwave; RT, room temperature; mAb, monoclonal antibody.

### Evaluation of tissue staining results

#### Evaluation of HSP27, VEGF-A, VEGF165b and bFGF expression

All sections were subjectively screened in a light microscope (Olympus CX31) at magnifications x40 and x100 to determine areas containing at least 50% tumor tissue; areas of ulceration or necrosis within the tumor were avoided. Subsequently, staining intensity and the proportion of positive tumor cells and endothelial cells (ECs) within the area of each high power field (HPF, x400) were recorded using a semi-quantitative grading. Staining intensity was defined as absent (0), weak (1), moderate (2) or strong (3). The proportion was rated as “no positive tumor cells” (0), “less than 10% positive tumor cells” (1), “10–50% positive tumor cells” (2) or “more than 50% positive tumor cells” (3). The staining index (SI) is the product of intensity and area (range 0–9) [9]; SI was used to determine cytoplasmic staining of VEGF-A and HSP27 and to record cytoplasmic and nuclear staining of VEGF165b and bFGF. The SI was evaluated by two observers (CS, OS) blinded for response data.

### Evaluation of microvessel density and glomeruloid microvascular proliferations

Assessment of MVD and pMVD was done after dual staining (F-VIII/Ki67). F-VIII positive ECs and microvessels were counted in three HPFs to assess MVD in primary tumors and metastases. Sections were first screened at lower magnification (x40 and x100) for selection of MVD hot-spot areas defined by high intensity of F-VIII [11, 26]. Areas of ulceration or necrosis within the tumor were avoided. Microvessels with co-expression of Ki67 in the nucleus and F-VIII in the cytoplasm of ECs were defined as proliferating vessels. The pMVD was recorded in the same three HPFs chosen for assessment of MVD. Ki67 positive nuclei within the lumen or outside ECs were excluded. Both MVD and pMVD were reported as microvessel per mm^2^. In addition, VPI was calculated as the ratio between pMVD and MVD (% of 100) [27].

Assessment of GMP was done after staining with F-VIII and hematoxylin counterstain. GMPs were defined as the presence of focal glomerulus-like aggregates of related multilayered F-VIII positive ECs with a minimum number of 15 cells. After screening the tumor at lower magnification, GMPs were registered by x100 magnification in a maximum of four consecutive HPFs within the area of highest density. Presence or absence of GMPs was finally reported [28].

### Enzyme-linked immunosorbent assay (ELISA)

ELISA was performed according to the manufacturer`s instructions for HSP27 (Enzo Life Science, ADI-EKS-500) and for bFGF (R&D systems, DFB50) in serum samples as well as for VEGF-A_165_ (R&D systems, DVE00) in plasma and serum samples. Some samples were diluted to fit within the absorbance of the HSP27-standard curve. Samples were run in duplicates. Referring to the user`s manual, the MDD was less than 0.39 ng/ml for HSP27, 3.0 pg/ml for bFGF and 9.0 pg/ml for VEGF. Results below these limits were considered to be zero.

### Statistics

Statistical analyses were performed with SPSS, version 22 (SPSS Inc., Chicago, IL). For comparison of two categorical variables, Pearson`s chi-square test was used. Since not all data followed normal distribution, non-parametric tests were used for all analyses. Continuous and ordinal variables were assessed by the Mann-Whitney U Test (MWT) or the Kruskal-Wallis Test (KWT). Intratumoral protein expression, MVD or pMVD in primary tumors and metastases as well as blood concentrations were independent variables when MWT was performed to calculate the association with response or treatment line. Kruskal-Wallis test was used to calculate the association between Breslow thickness and protein expression in primary melanomas (independent variable). The association of various proteins and angiogenic factors between primary tumors and metastases were calculated by the paired Wilcoxon Test (pWT) by the assumption that samples from the primary melanoma and the metastasis are matched pairs. Strength and direction of correlations between two interval scaled continuous or ordinal variables or one of each type were calculated by Spearman`s rho correlation. The level of significance was defined as p < 0.05. When continuous variables were categorized, the median was used as cut-point if no other cut-point is defined. The statistical analyses of this study were performed with pre-specified hypotheses for a limited number of intratumoral proteins related to VEGF-A associated angiogenesis. Thus, we did not correct for multiple testing.

## Results

### Tissue expression of angiogenic factors

#### HSP27

**Fig 1:**
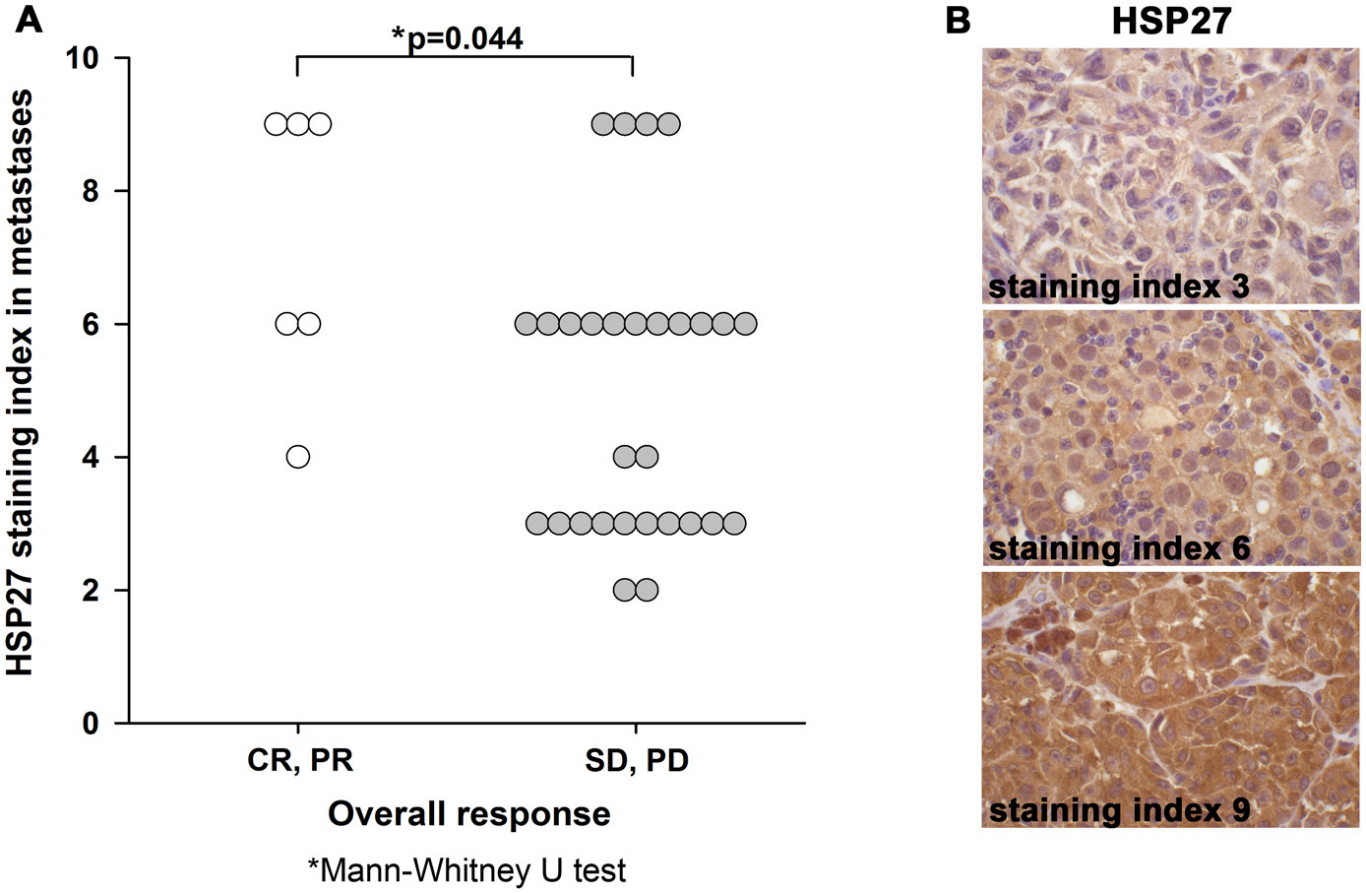
HSP27 expression in metastases predicts overall response to treatment with bevacizumab. (A) HSP27 expression in metastases grouped by treatment response. CR: complete clinical response, PR: partial response, SD: stable disease, PD: progressive disease. (B) Examples for low, moderate and high staining index for HSP27. Original magnification x630.

HSP27 was expressed in the cytoplasm of melanoma cells in all primary tumors and metastases. The median staining index (SI) was 6 for both primary tumors and metastases (p = 0.74; paired Wilcoxon Test, (pWT)). Strong HSP27 expression in metastases was significantly associated with overall response (OR) to treatment with bevacizumab monotherapy. Median HSP27 SI for patients with OR was 7.5 compared to a median SI of 6 in patients with stable disease (SD) or progressive disease (PD) (p = 0.044; Mann-Whitney U Test (MWT); Fig 1A and 1B; Table 2). Although some patients with HSP27 overexpression did not respond to treatment, no one with low or absent HSP27 expression belonged to the OR-group. There was also a trend for an association between HSP27 expression and OR in primary tumors (p = 0.097; MWT), and HSP27 staining in primary melanomas was significantly associated with CB from bevacizumab treatment (p = 0.046; MWT, S1 Table). Histologic features of the primary tumors were not associated with HSP27 expression, nor with response. Median HSP27 staining in metastases was significantly stronger within the group treated in first line with bevacizumab compared to second line treatment (median SI 6 vs 3, p = 0.008; MWT, S2 Table).

**Table 2:** Descriptive data for HSP27 expression in metastases.

HSP27 expression in metastases	Overall response (OR)	No OR	Clinical benefit (CB)	No CB
**Mean SI**^a^ **+/-SEM**^b^	**7.2 +/- 0.9**	**5.0 +/- 0.4**	**6.3 +/- 0.8**	**4.9 +/- 0.4**
Median SI*	**7.5**	**6**	**6**	**5**
**Minimum SI**	**4**	**2**	**2**	**2**
**Maximum SI**	**9**	**9**	**9**	**9**
**Number of patients**	**6**	**29**	**11**	**24**

* p = 0.044 (OR), p = 0.15 (CB);

Mann-Whitney U Test.

^a:^ Staining index (SI);

^b:^ Standard error of mean (SEM).

#### VEGF-A

**Fig 2:**
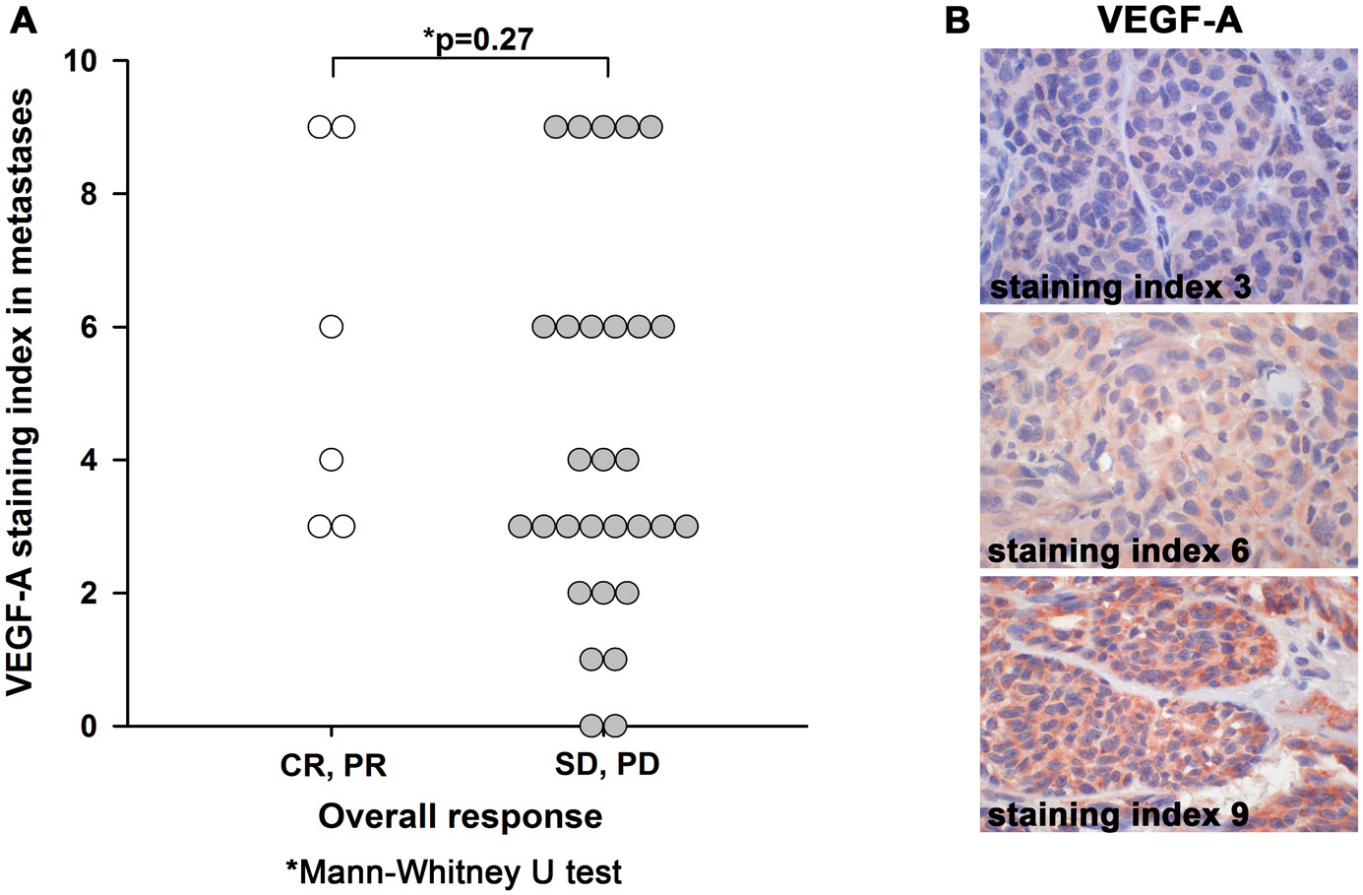
VEGF-A expression in metastases is not associated with overall response to bevacizumab. (A) VEGF-A expression in metastases grouped by treatment response. CR: complete clinical response, PR: partial response, SD: stable disease, PD: progressive disease. (B) Examples for low, moderate and high staining index for VEGF-A. Original magnification x630.

Cytoplasmic expression of VEGF-A was assessed separately in melanoma cells and ECs in primary tumors and metastases. All primary melanomas, except one, expressed VEGF-A in tumor cells (median SI = 5), and most metastases as well (33 of 35; median SI = 4). However, the difference in VEGF-A SI between primary tumors and metastases was not significant (p = 0.71; pWT). VEGF-A expression in metastases was neither significantly correlated to OR (Fig 2A and 2B) nor to CB (Table 3). Treatment outcome did not depend on VEGF-A expression in primary tumors (S3 Table). VEGF-A expression in metastases did not correlate significantly with HSP27 expression in metastases. VEGF-A expression in primary tumors was not associated with any of the histologic features. Median VEGF-A expression was significantly stronger in metastases from patients treated with bevacizumab in first line compared to patients treated in second line (median SI 6 vs 3 p = 0.016; MWT, S4 Table).

**Table 3:** Descriptive data for VEGF-A expression in metastases.

VEGF-A expression in metastases	Overall response (OR)	No OR	Clinical benefit (CB)	No CB
**Mean SI**^a^ **+/- SEM**^b^	**5.7 +/- 1.1**	**4.3 +/- 0.5**	**5.4 +/- 0.8**	**4.2 +/- 0.6**
Median SI*	**5**	**3**	**6**	**3**
**Minimum SI**	**3**	**0**	**2**	**0**
**Maximum SI**	**9**	**9**	**9**	**9**
**Number of patients**	**6**	**29**	**11**	**24**

^a:^ Staining index (SI);

^b:^ Standard error of mean (SEM)

* p = 0.27 (OR), p = 0.30 (CB);

Mann-Whitney U Test.

VEGF-A expression in tumor associated ECs was present in 21/32 (66%) of primary tumors and 15/35 (43%) of metastases. There was no association between EC-VEGF-A expression (primary melanoma or metastases) and response to treatment.

#### VEGF165b

Both cytoplasmic and nuclear expression of VEGF165b was observed in tumor cells of all primary melanomas and metastases. The median cytoplasmic SI was similar in primary tumors and metastases (p = 0.22; pWT) but the nuclear SI was significantly lower in metastases (p = 0.045; pWT, S1 Fig). The expression level of VEGF165b in primary tumors or metastases was not associated with response to treatment. No associations with histologic features or expression of VEGF-A were observed.

#### bFGF

Cytoplasmic bFGF expression was observed in 31/32 primary melanomas and all metastases; nuclear bFGF expression was seen in 30/32 primary melanomas and 28/35 metastases. The median nuclear SI was similar in primary tumors and metastases (p = 0.174; pWT) but the cytoplasmic SI was significantly lower in metastases (p = 0.030; pWT, S2 Fig). There was no association between expression of bFGF in primary melanomas or metastases and response to treatment. Regarding histologic features, primary tumors with a mitotic rate < 1/mm^2^ showed stronger cytoplasmic and nuclear expression of bFGF (p = 0.03 and p = 0.02; MWT), and strong nuclear expression was associated with the absence of ulceration and lower Breslow thickness (p = 0.014; MWT; p = 0.021; Kruskal-Wallis test (KWT)).

#### MVD, pMVD, VPI and GMP

**Fig 3:**
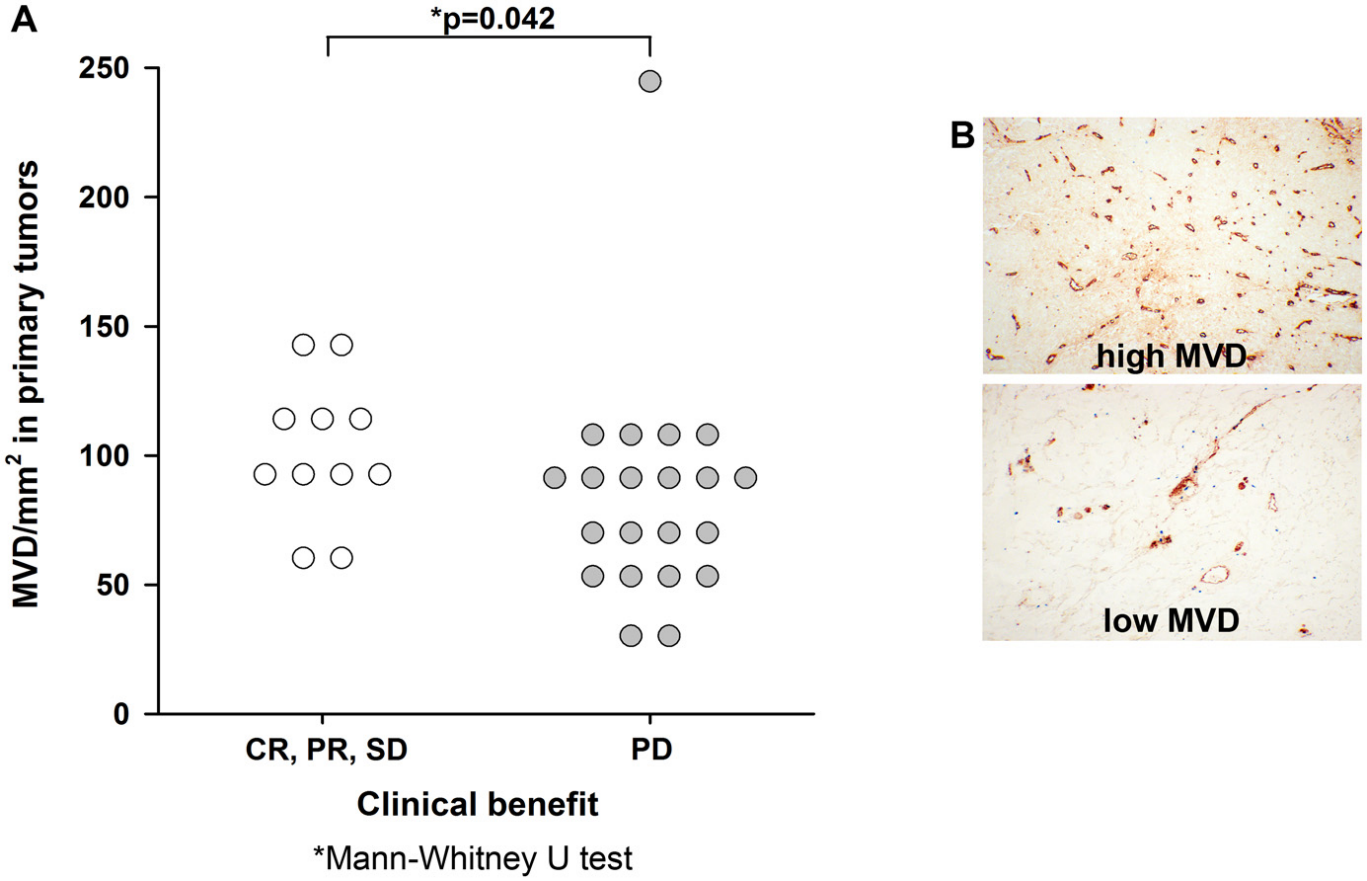
High microvessel density (MVD) in primary tumors predicts clinical benefit to treatment with bevacizumab. (A) MVD in primary melanomas grouped by treatment response. Median MVD in primary melanomas is significantly higher in patients with clinical benefit. CR: complete clinical response, PR: partial response, SD: stable disease, PD: progressive disease (B) Immunohistochemical staining showing high and low MVD. Original magnification x100.

Quantification of angiogenesis markers MVD, pMVD and GMP was based on immunohistochemical staining of tumor associated ECs and proliferating ECs. Median MVD was 89/mm^2^ (mean 90/mm^2^) in primary melanomas and 108/mm^2^ (mean 107/mm^2^) in metastases; thus, MVD was significantly higher in metastases than in primary melanomas (p = 0.031; pWT). High MVD in primary tumors predicted CB to treatment with a median MVD of 103/mm^2^ in responders vs. 83/mm^2^ in non-responders respectively (p = 0.042; MWT), (Fig 3A and 3B; S3 Fig). No significant association was present between MVD in metastases and treatment response. However, high MVD in metastases was correlated to strong VEGF-A expression (p = 0.025; r = 0.39; Spearman).

Median pMVD was significant lower in primary melanomas (3.6/mm^2^; mean 5.8/mm^2^) than in metastases (8.9/mm^2^; mean 9.9/mm^2^) (p = 0.01; pWT). There was no association between pMVD and treatment response. Also, median VPI was significantly lower in primary melanomas (5.1; mean 6.1) than in metastases (7.8; mean 10.4) (p = 0.035; pWT). VPI was not associated with treatment response. In primary tumors, strong VEGF-A expression was correlated to higher levels of pMVD and VPI (p = 0.028 and p = 0.027; r = 0.39 for both; Spearman).

GMPs were present in 8/32 (25%) of primary melanomas and 4/34 (12%) of metastases. Presence of GMPs (primary melanomas or metastases) did not predict response to treatment.

### Concentrations of angiogenic factors in blood samples

#### HSP27 concentration in serum samples

All measurements of HSP27 concentration in serum (sHSP27) were above minimal detectable dose (MDD) (median 7.07 ng/ml). sHSP27 concentration was not associated with response to treatment (S11 Table). Notably, there was no association between sHSP27 concentration and tissue expression of HSP27 in metastases, nor with VEGF-A tissue expression. High sHSP27 was correlated with high VEGF-A in plasma and serum as well as with high bFGF in serum (p = 0.004; r = 0.52; p = 0.038; r = 0.39 and p<0.001; r = 0.76; Spearman, respectively). Notably, median sHSP27 was nine fold higher in patients treated with bevacizumab in second line compared with first line (p<0.001; MWT, S12 Table). Further, high sHSP27 was related to high LDH at the time of inclusion (p = 0.039; r = 0.39; Spearman). sHSP27 was not correlated to MVD, pMVD, VPI or GMP in metastases.

#### VEGF-A concentration in serum and plasma samples

The VEGF-A concentration was above MDD in all serum samples (sVEGF, median 345 pg/ml) and in 23/29 plasma samples (pVEGF, median 52 pg/ml). High VEGF-A concentration in serum was significantly correlated to high VEGF-A concentration in plasma (p<0.001; r = 0.75; Spearman). VEGF concentrations in serum or plasma were not associated with response to treatment (S11 Table). Furthermore, there was no correlation to VEGF-A expression in metastases. Median pVEGF was almost six fold higher in patients treated with bevacizumab in second line compared with first line (p = 0.017; MWT, S12 Table). High sVEGF and pVEGF were associated with high bFGF in serum (p = 0.029; r = 0.41 and p = 0.004; r = 0.52; Spearman) and high baseline LDH (p = 0.018 and p = 0.032; r = 0.4; for both, Spearman). There were no significant correlations between VEGF concentrations in blood samples and tissue based angiogenesis markers (MVD, pMVD, VPI, GMP) in metastases.

#### bFGF in serum

The bFGF concentration in serum (s-bFGF) was above MDD in 21/28 samples (median 7.8 pg/ml). The concentration of bFGF was not associated with response to treatment (S11 Table). High s-bFGF was correlated to high sHSP27 and high plasma and serum VEGF (see above). bFGF concentration in serum was not associated with expression of any tissue markers in metastases including MVD, pMVD, VPI or GMP status. Notably, median bFGF was significantly higher in serum samples from patients treated in second line (p = 0.002; MWT, S12 Table).

## Discussion

Several attempts to identify biomarkers of response to bevacizumab or other anti-angiogenic drugs were recently reviewed by Lambrechts et al. [19]. Validation of potential predictive biomarkers for anti-angiogenic treatment is important to avoid severe side-effects among patients without benefit, and to define treatment indications for optimized and personalized medicine. Since angiogenesis is a complex and dynamic process involving many different growth factors, cytokines and interactions with the stroma [31], there is a challenge to identify individual predictive markers.

Multiple angiogenic factors have been investigated in randomized trials for various cancers [19]. Still, none are currently established as predictive markers. The role of VEGF-A and different VEGF-A SNPs as response predictors has been investigated in clinical trials of bevacizumab. The results are inconsistent, and although associations to improved progression free [32] or overall survival [33] were reported in some trials, none of these markers have been firmly validated [19, 34].

So far, treatment induced hypertension appears to predict clinical benefit in patients treated with anti-angiogenic drugs as reported in metastatic renal cell cancer [35] and metastatic melanoma [14]. In contrast, early hypertension did not predict treatment response in metastatic colorectal cancer when bevacizumab was given in combination with chemotherapy [36].

Here, as a novel finding, strong tissue expression of HSP27 in melanoma metastases was associated with overall response (OR) to bevacizumab monotherapy. Strong expression of HSP27 in metastases characterized all patients with OR, and no patients with weak or absent HSP27 expression were among the responders. Other studies have identified strong HSP27 expression as a prognostic marker for poor outcome in various solid tumors [37–39], and increased intratumoral expression is associated with more advanced tumor stages in ovarian cancer [40]. Furthermore, strong HSP27 expression in vitro and in vivo is associated with resistance to chemotherapy and its downregulation increased sensitivity to treatment [40–43]. HSP27, which is phosphorylated and activated in cells under stressful conditions like hypoxia, is involved in angiogenesis by regulating expression and secretion of VEGF as previously reported [24, 25]. Straume et al. investigated the mechanistic role of HSP27 in angiogenesis in various models. Knockdown of HSP27 in an angiogenic breast cancer cell line resulted in phenotypic non-angiogenic and microscopic xenograft tumors. Gene expression analysis showed reduced expression of HSP27 as well as of VEGF-A and bFGF [24]. In addition, strong expression of HSP27 was associated with decreased survival in melanoma and breast cancer [24]. Our present finding that strong expression of HSP27 in melanoma metastases is associated with response to anti-VEGF therapy, is in support of HSP27 being a negative prognostic factor and a predictive indicator for treatment response. The reason for this might be that increased HSP27 expression identifies an aggressive melanoma phenotype with an active VEGF dependent angiogenesis that is more sensitive to anti-VEGF treatment.

Although we found a strong correlation between sHSP27 and pVEGF, sVEGF as well as s-bFGF in blood samples taken before treatment with bevacizumab, there was no association between tissue expression of HSP27 in metastases and sHSP27, similar to a previous study on breast cancer [44], and no relationship between blood levels of these factors and response to treatment.

Apart from the significant correlation between high MVD in primary tumors and clinical benefit, we observed no significant associations between VEGF, bFGF (in tissues or blood samples), VEGF165b or tissue based angiogenesis markers and response to treatment. This is in line with the disappointing conclusions from multiple studies of anti-angiogenesis treatment and biomarkers in various cancers [19, 34, 45].

The associations of all assessed intratumoral proteins and angiogenic factors between the primary tumors and metastases were investigated. The median protein expression was similar in primary tumors and metastases except for cytoplasmic bFGF and nuclear VEGF165b. Contrary, the median of MVD, pMVD and VPI was significantly higher in metastases. Based on these findings, a biopsy of the metastatic lesion should be used for investigations of predictive factors.

There was a lack of consistent associations between tissue expression of angiogenesis markers and their levels observed in plasma or serum samples. These findings might be explained by changing tumor-stroma interactions and heterogeneity in primary tumors and metastases, as well as the possibility of clonal evolution and tumor progression in metastatic tumors. Under physiologic angiogenesis, *i*.*e*. during wound healing, an inverse relation between local and systemic levels of angiogenic factors has been repeatedly observed [46, 47]. These changes might in part explain the challenges in robust quantification of dynamic growth factors, especially ligands like VEGF and bFGF, during cancer progression and treatment.

Since VEGF-A is a key player in angiogenesis and a specific treatment target on this trial, we also looked at associations between VEGF-A and other markers. VEGF-A tissue expression was significantly associated with microvessel proliferation (pMVD and VPI) in primary melanomas, and with overall microvessel density (MVD) in metastases. Taken together, our findings support an important role of VEGF-A in melanoma angiogenesis and progression as previously indicated [28]. Nevertheless, none of these angiogenesis markers were associated with blood levels of VEGF. Our results are in line with findings by Byrne et al. who reported that VEGF expression in primary breast cancer patients was significantly associated with MVD but not with VEGF concentration in platelet-depleted plasma [48].

The intratumoral expression of some proteins as well as their blood concentrations differed significantly between the patients treated with bevacizumab in first line and second line. Median HSP27 expression in tumor cells in metastases was significantly reduced in patients treated with DTIC in first line followed by bevacizumab when compared to the group of patients treated with bevacizumab in first line. Most responders were found in the latter group [14]. The reason why HSP27 expression was significantly reduced in patients treated with DTIC in first line is not clear. Experimental studies have shown that exposure to both single and repeated doses of DTIC could select for a more aggressive melanoma phenotype through induction of VEGF and interleukin 8 by mechanisms other than HSP27 [49]. Thus, pretreatment with DTIC in first line might have selected a more complex angiogenic phenotype, more resistant to the specific inhibition of VEGF-A with bevacizumab.

Contrary, serum HSP27 was nine fold higher in patients treated with DTIC in first line followed by bevacizumab. Although, the mechanism of HSP27 secretion is not yet completely understood, release from necrotic cells after exposure to chemotherapy could contribute to increased extracellular levels of HSP27 [50]. Similarly, to sHSP27, the concentrations of pVEGF and s-bFGF were significantly higher in patients treated with DTIC in first line. This is again pointing to the inverse relation between local and systemic levels of angiogenic factors, and suggests that the increased systemic levels of these factors might have other sources than the tumor cells [13].

There are limitations to the present study. One is the low number of patients included, with a relative lack of statistical power. Another problem is the limited amount of tissue available for analysis especially from some metastatic lesions. It is well known that tumor angiogenesis is not evenly distributed and tends to occur in hot-spot areas. The sampling bias might therefore reduce sensitivity and might have impact on the angiogenesis quantification by markers like MVD and pMVD, as well as on the representativeness of intratumoral protein expression, especially regarding small samples from metastatic lesions. However, metastatic melanoma tissue was sampled in accordance with contemporary practice. It remains to be investigated if such tissue samples are sufficiently robust for predictive purposes. On the other hand, since no chemotherapy was given concomitantly, the findings are based on the effects of a single drug. Another advantage is the availability of matched primary tumors and metastases as well as serum and plasma samples. In addition, histologic features of the primary tumors as well as clinical features and follow-up data, including response information, were available. Nevertheless, because of the low number of patients, limited amount of tissue, in some cases of core needle biopsies, as well as the lack of a randomized control group, the results must be interpreted carefully.

In conclusion, our data indicate that strong expression of HSP27 protein in melanoma metastases predicts overall response to bevacizumab monotherapy in patients with metastatic melanoma. In contrast, multiple other angiogenesis markers, examined in tissues and blood samples, showed no relationship with treatment response. Further randomized studies are necessary to validate our findings.

## Supporting Information

S1 Fig Nuclear VEGF165b expression in primary melanomas and metastases. (TIF)

S2 Fig Nuclear bFGF expression in primary melanomas and metastases. (TIF)

S3 Fig Microvessel density in primary melanomas according to treatment response. (TIF)

S4 Fig Proliferating microvessels and glomeruloid microvascular proliferation (GMP). Proliferating vessels* show positive cytoplasmic staining for Factor VIII (red) and positive nuclear staining for Ki67 (blue). Original magnification x400. GMP: Focal glomerulus-like aggregates of closely associated and multilayered Factor VIII positive endothelial cells. Original magnification x630.
(TIF)

S1 Table Descriptive data for HSP27 expression in primary tumors. (DOCX)

S2 Table Descriptive data for HSP27 expression in metastases according to line of treatment. (DOCX)

S3 Table Descriptive data for VEGF-A expression in primary tumors. (DOCX)

S4 Table Descriptive data for VEGF-A expression in metastases according to line of treatment. (DOCX)

S5 Table Descriptive data for microvessel density (MVD) in primary tumors. (DOCX)

S6 Table Descriptive data for microvessel density (MVD) in metastases. (DOCX)

S7 Table Descriptive data for proliferating microvessel density (pMVD) in primary tumors. (DOCX)

S8 Table Descriptive data for proliferating microvessel density (pMVD) in metastases. (DOCX)

S9 Table Descriptive data for vascular proliferation index (VPI) in primary tumors. (DOCX)

S10 Table Descriptive data for vascular proliferation index (VPI) in metastases. (DOCX)

S11 Table Concentrations of HSP27, VEGF-A and bFGF in blood samples according to overall response. (DOCX)

S12 Table Concentrations of HSP27, VEGF-A and bFGF in blood samples according to line of treatment. (DOCX)
