# Supplementary figures and images for: Expression of Heat Shock Protein 27 in Melanoma Metastases Is Associated with Overall Response to Bevacizumab Monotherapy: Analyses of Predictive Markers in a Clinical Phase II Study
Source: PLoS One. 2016 May 11;11(5):e0155242. doi: 10.1371/journal.pone.0155242 (PMC4864228; doi:10.1371/journal.pone.0155242)

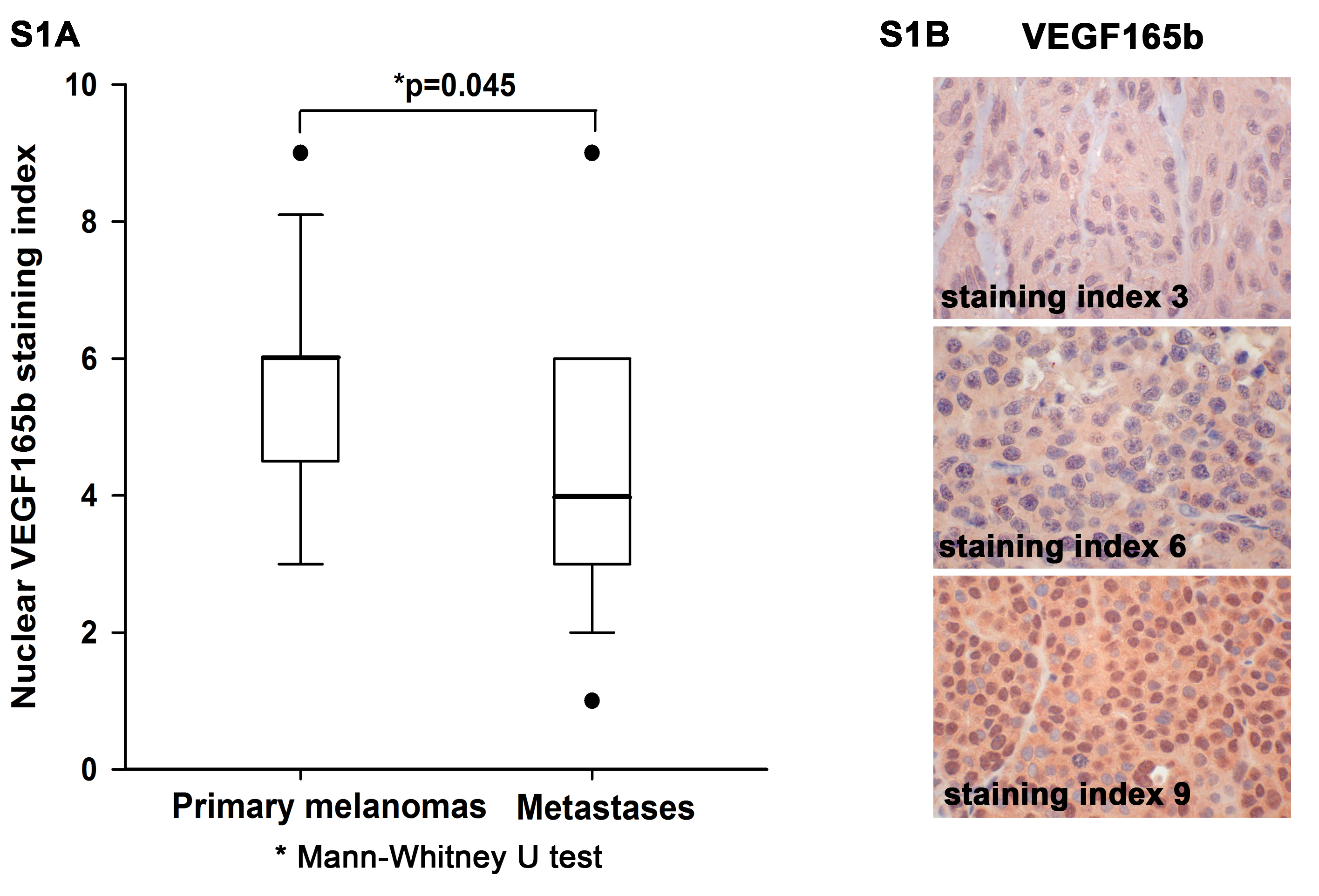

Supplement: S1 Fig — (TIF) [file pone.0155242.s001.tif]

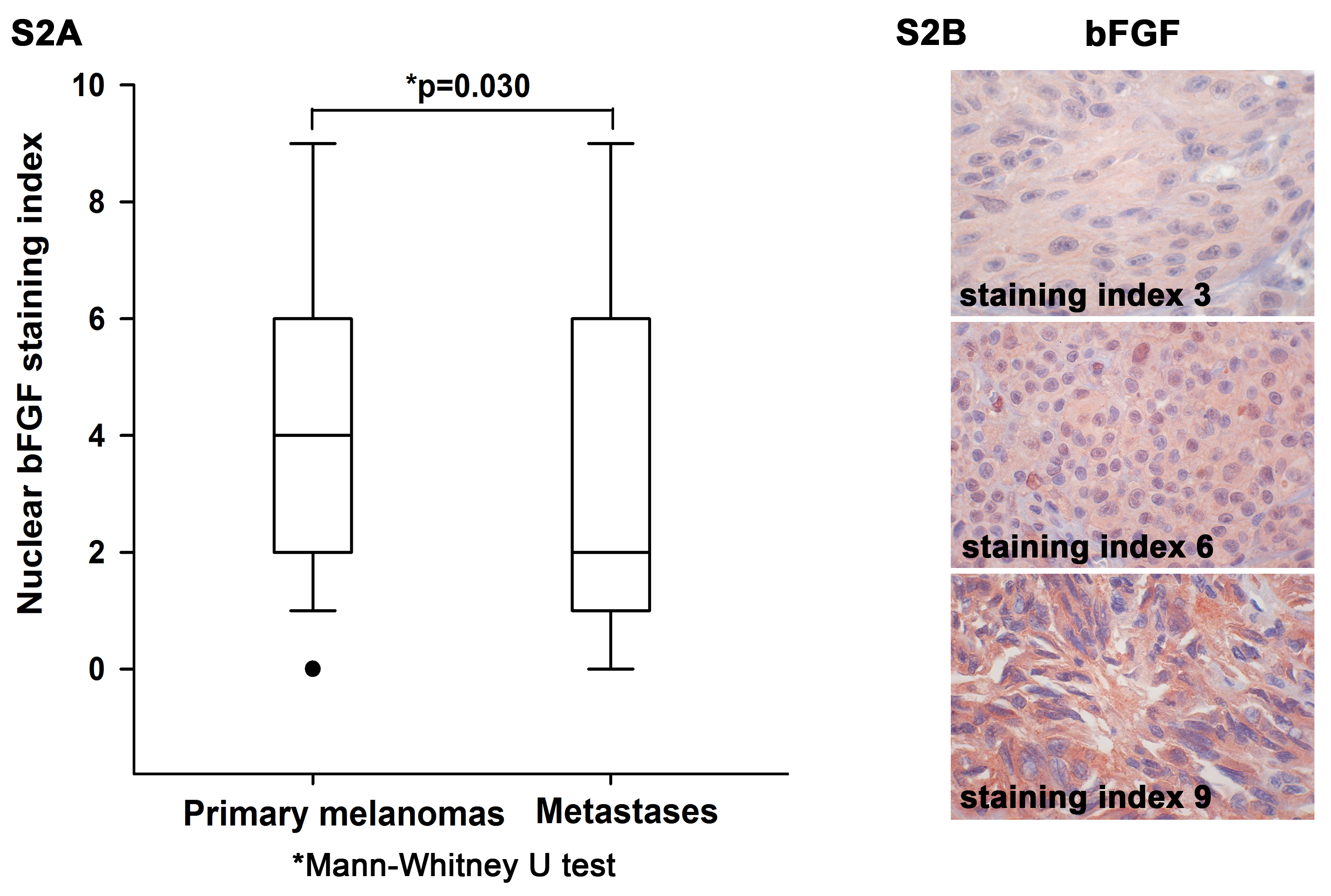

Supplement: S2 Fig — (TIF) [file pone.0155242.s002.tif]

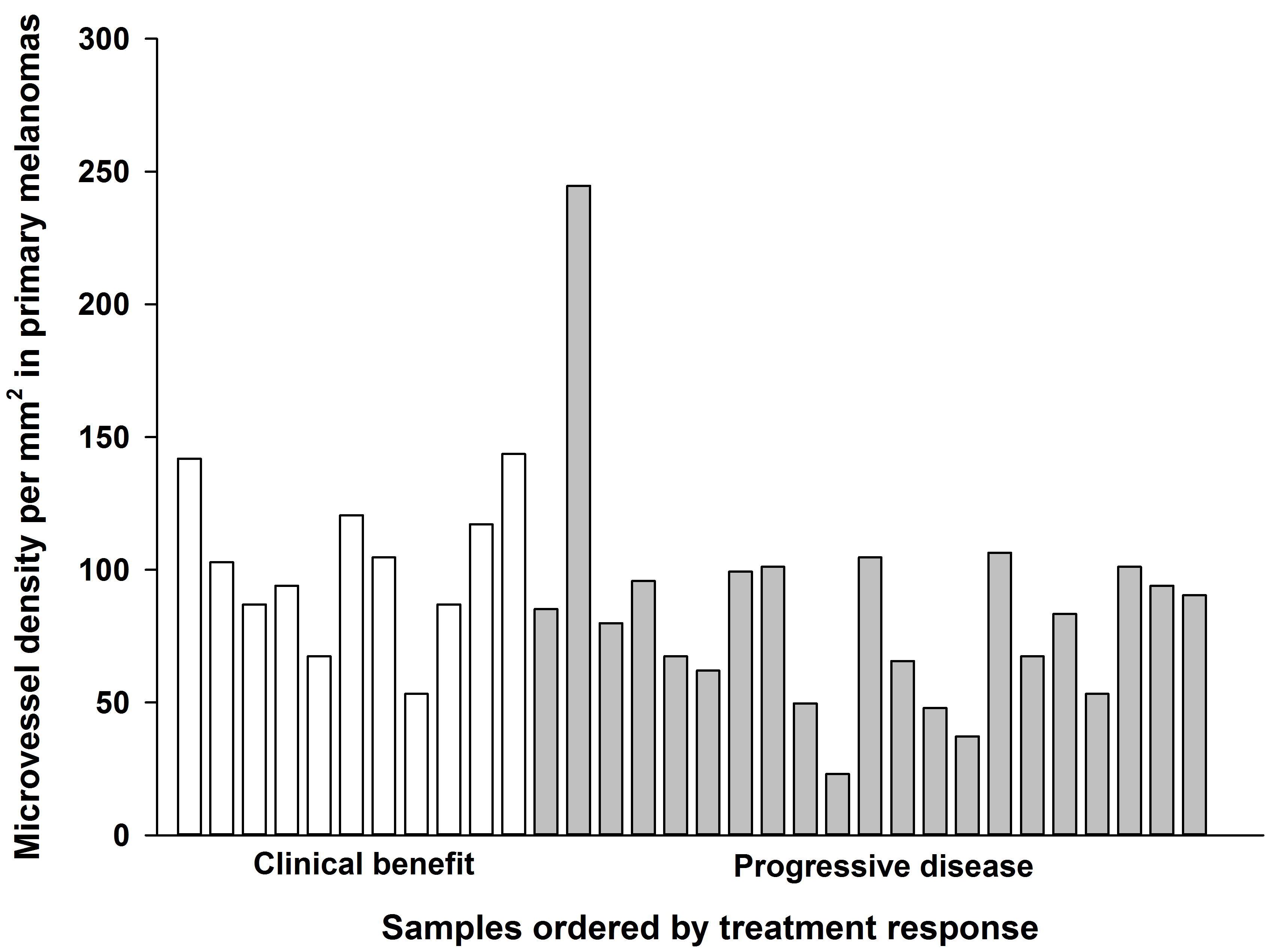

Supplement: S3 Fig — (TIF) [file pone.0155242.s003.tif]

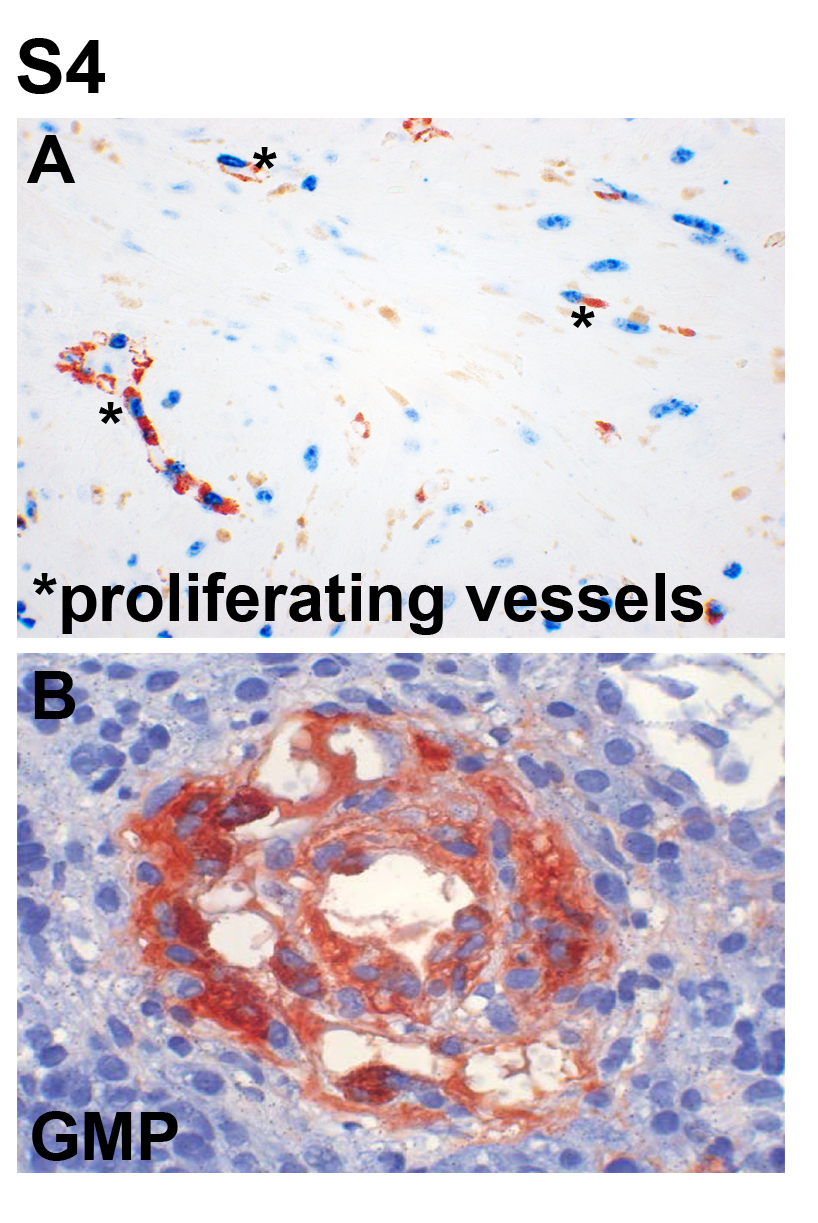

Supplement: S4 Fig — Proliferating vessels* show positive cytoplasmic staining for Factor VIII (red) and positive nuclear staining for Ki67 (blue). Original magnification x400. GMP: Focal glomerulus-like aggregates of closely associated and multilayered Factor VIII positive endothelial cells. Original magnification x630. (TIF) [file pone.0155242.s004.tif]
