# Supplementary material for: Expression of Heat Shock Protein 27 in Melanoma Metastases Is Associated with Overall Response to Bevacizumab Monotherapy: Analyses of Predictive Markers in a Clinical Phase II Study
Source: PLoS One. 2016 May 11;11(5):e0155242. doi: 10.1371/journal.pone.0155242 (PMC4864228; doi:10.1371/journal.pone.0155242)
Supplement: S1 Table — (DOCX) [file pone.0155242.s005.docx]

**S1 Table. Descriptive data for HSP27 expression in primary tumors**

| **HSP27 expression in primary tumors** | **Overall response (OR)** | **No OR** | **Clinical benefit (CB)** | **No CB** |
| --- | --- | --- | --- | --- |
| **Mean SI^a^ +/- SEM^b^** | **6.5 +/- 0.5** | **5 +/- 0.3** | **6.3 +/- 0.5** | **4.8 +/- 0.3** |
| **Median SI^*^** | **6** | **6** | **6** | **6** |
| **Minimum SI** | **6** | **2** | **3** | **2** |
| **Maximum SI** | **9** | **9** | **9** | **6** |
| **Number of patients** | **6** | **26** | **11** | **21** |

a: Staining index (SI); b: Standard error of mean (SEM)

* p=0.097 (OR), p=0.046 (CB); Mann-Whitney U Test.
