# Supplementary material for: Expression of Heat Shock Protein 27 in Melanoma Metastases Is Associated with Overall Response to Bevacizumab Monotherapy: Analyses of Predictive Markers in a Clinical Phase II Study
Source: PLoS One. 2016 May 11;11(5):e0155242. doi: 10.1371/journal.pone.0155242 (PMC4864228; doi:10.1371/journal.pone.0155242)
Supplement: S2 Table — (DOCX) [file pone.0155242.s006.docx]

**S2 Table. Descriptive data for HSP27 expression in**

**metastases** **according to line of treatment**

| **HSP27 expression in metastases** | **1st line bevacizumab** | **2nd line bevacizumab** |
| --- | --- | --- |
| **Mean SI^a^ +/- SEM^b^** | **6.2 +/- 0.5** | **4.1 +/- 0.5** |
| **Median SI^*^** | **6** | **3** |
| **Minimum SI** | **3** | **2** |
| **Maximum SI** | **9** | **9** |
| **Number of patients** | **21** | **14** |

a: Staining index (SI); b: Standard error of mean (SEM)

* p=0.008; Mann-Whitney U Test
