# Supplementary material for: Expression of Heat Shock Protein 27 in Melanoma Metastases Is Associated with Overall Response to Bevacizumab Monotherapy: Analyses of Predictive Markers in a Clinical Phase II Study
Source: PLoS One. 2016 May 11;11(5):e0155242. doi: 10.1371/journal.pone.0155242 (PMC4864228; doi:10.1371/journal.pone.0155242)
Supplement: S5 Table — (DOCX) [file pone.0155242.s009.docx]

**S5 Table. Descriptive data for microvessel density (MVD) in primary tumors**

| **MVD in primary tumor** | **Overall response (OR)** | **No OR** | **Clinical benefit (CB)** | **No CB** |
| --- | --- | --- | --- | --- |
| **Mean MVD +/- SEM^a^** | **89.2 +/- 10.3** | **90.1 +/- 8.4** | **101.7 +/- 8.5** | **83.8 +/- 9.6** |
| **Median MVD^*^** | **94.9** | **88.7** | **102.8** | **83.3** |
| **Minimum MVD** | **53.2** | **23.1** | **53.2** | **23.1** |
| **Maximum MVD** | **120.6** | **244.7** | **143.6** | **244.7** |
| **Number of patients** | **6** | **26** | **11** | **21** |

a: Standard error of mean (SEM); * p=0.62 (OR), p=0.042 (CB); Mann-Whitney U Test.
