# Supplementary material for: Expression of Heat Shock Protein 27 in Melanoma Metastases Is Associated with Overall Response to Bevacizumab Monotherapy: Analyses of Predictive Markers in a Clinical Phase II Study
Source: PLoS One. 2016 May 11;11(5):e0155242. doi: 10.1371/journal.pone.0155242 (PMC4864228; doi:10.1371/journal.pone.0155242)
Supplement: S6 Table — (DOCX) [file pone.0155242.s010.docx]

**S6 Table. Descriptive data for microvessel density (MVD) in metastases**

| **MVD in metastases** | **Overall response (OR)** | **No OR** | **Clinical benefit (CB)** | **No CB** |
| --- | --- | --- | --- | --- |
| **Mean MVD +/- SEM^a^** | **104.6 +/- 18.2** | **107.1 +/- 10.4** | **93.4 +/-14.7** | **112.4 +/- 11.2** |
| **Median MVD^*^** | **97.5** | **108.2** | **86.0** | **115.3** |
| **Minimum MVD** | **60.3** | **24.8** | **28.4** | **24.8** |
| **Maximum MVD** | **154.3** | **212.8** | **154.3** | **212.8** |
| **Number of patients** | **6** | **27** | **10** | **23** |

a: Standard error of mean (SEM); * p=0.98 (OR), p=0.38 (CB); Mann-Whitney U Test.
