# Supplementary material for: Expression of Heat Shock Protein 27 in Melanoma Metastases Is Associated with Overall Response to Bevacizumab Monotherapy: Analyses of Predictive Markers in a Clinical Phase II Study
Source: PLoS One. 2016 May 11;11(5):e0155242. doi: 10.1371/journal.pone.0155242 (PMC4864228; doi:10.1371/journal.pone.0155242)
Supplement: S7 Table — (DOCX) [file pone.0155242.s011.docx]

**S7 Table. Descriptive data for proliferating microvessel density (pMVD) in primary tumors**

| **pMVD in primary tumor** | **Overall response (OR)** | **No OR** | **Clinical benefit (CB)** | **No CB** |
| --- | --- | --- | --- | --- |
| **Mean pMVD +/- SEM^a^** | **5.0 +/- 2.0** | **6.0 +/- 1.4** | **5.2 +/-1.4** | **6.2 +/- 1.6** |
| **Median pMVD^*^** | **4.4** | **3.5** | **3.6** | **3.6** |
| **Minimum pMVD** | **0** | **0** | **0** | **0** |
| **Maximum pMVD** | **14.2** | **30.1** | **14.2** | **30.1** |
| **Number of patients** | **6** | **26** | **11** | **21** |

a: Standard error of mean (SEM); * p=0.98 (OR), p=0.85 (CB); Mann-Whitney U Test.
