# Supplementary material for: Expression of Heat Shock Protein 27 in Melanoma Metastases Is Associated with Overall Response to Bevacizumab Monotherapy: Analyses of Predictive Markers in a Clinical Phase II Study
Source: PLoS One. 2016 May 11;11(5):e0155242. doi: 10.1371/journal.pone.0155242 (PMC4864228; doi:10.1371/journal.pone.0155242)
Supplement: S8 Table — (DOCX) [file pone.0155242.s012.docx]

**S8 Table. Descriptive data for proliferating microvessel density (pMVD) in metastases**

| **pMVD in metastases** | **Overall response (OR)** | **No OR** | **Clinical benefit (CB)** | **No CB** |
| --- | --- | --- | --- | --- |
| **Mean pMVD +/- SEM^a^** | **15.7 +/- 5.0** | **8.6 +/- 1.5** | **9.8 +/- 3.8** | **9.9 +/- 1.6** |
| **Median pMVD^*^** | **11.53** | **7.1** | **5.3** | **8.9** |
| **Minimum pMVD** | **5.3** | **0** | **0** | **0** |
| **Maximum pMVD** | **35.5** | **31.9** | **35.5** | **31.9** |
| **Number of patients** | **6** | **27** | **10** | **23** |

a: Standard error of mean (SEM); * p=0.16 (OR), p=0.48 (CB); Mann-Whitney U Test.
