# Supplementary material for: Expression of Heat Shock Protein 27 in Melanoma Metastases Is Associated with Overall Response to Bevacizumab Monotherapy: Analyses of Predictive Markers in a Clinical Phase II Study
Source: PLoS One. 2016 May 11;11(5):e0155242. doi: 10.1371/journal.pone.0155242 (PMC4864228; doi:10.1371/journal.pone.0155242)
Supplement: S9 Table — (DOCX) [file pone.0155242.s013.docx]

**S9 Table. Descriptive data for vascular proliferation index (VPI) in primary tumors**

| **VPI in primary tumor** | **Overall response (OR)** | **No OR** | **Clinical benefit (CB)** | **No CB** |
| --- | --- | --- | --- | --- |
| **Mean VPI +/- SEM^a^** | **5.7 +/- 1.9** | **6.2 +/- 1.2** | **5.3 +/- 1.4** | **6.5 +/- 1.4** |
| **Median VPI^*^** | **4.6** | **5.1** | **4.1** | **5.3** |
| **Minimum VPI** | **0** | **0** | **0** | **0** |
| **Maximum VPI** | **13.6** | **21.6** | **13.6** | **21.6** |
| **Number of patients** | **6** | **26** | **11** | **21** |

a: Standard error of mean (SEM); * p=0.98 (OR), p=0.82 (CB); Mann-Whitney U Test.
