# Supplementary material for: Expression of Heat Shock Protein 27 in Melanoma Metastases Is Associated with Overall Response to Bevacizumab Monotherapy: Analyses of Predictive Markers in a Clinical Phase II Study
Source: PLoS One. 2016 May 11;11(5):e0155242. doi: 10.1371/journal.pone.0155242 (PMC4864228; doi:10.1371/journal.pone.0155242)
Supplement: S10 Table — (DOCX) [file pone.0155242.s014.docx]

**S10 Table. Descriptive data for vascular proliferation index (VPI) in metastases**

| **VPI in metastases** | **Overall response (OR)** | **No OR** | **Clinical benefit (CB)** | **No CB** |
| --- | --- | --- | --- | --- |
| **Mean VPI +/- SEM^a^** | **15.5 +/- 3.8** | **9.2 +/- 1.8** | **10.1 +/- 3.2** | **10.5 +/- 2.0** |
| **Median VPI^*^** | **15.4** | **7.6** | **7.8** | **7.8** |
| **Minimum VPI** | **3.5** | **0** | **0** | **0** |
| **Maximum VPI** | **29.0** | **46.2** | **29.0** | **46.2** |
| **Number of patients** | **6** | **27** | **10** | **23** |

a: Standard error of mean (SEM); * p=0.11 (OR), p=0.83 (CB); Mann-Whitney U Test.
