# Supplementary material for: Expression of Heat Shock Protein 27 in Melanoma Metastases Is Associated with Overall Response to Bevacizumab Monotherapy: Analyses of Predictive Markers in a Clinical Phase II Study
Source: PLoS One. 2016 May 11;11(5):e0155242. doi: 10.1371/journal.pone.0155242 (PMC4864228; doi:10.1371/journal.pone.0155242)
Supplement: S11 Table — (DOCX) [file pone.0155242.s015.docx]

**S11 Table. Concentrations of HSP27, VEGF-A and bFGF in blood samples according to overall response**

|  | **HSP27 (serum, ng/ml)** |  | **VEGF-A (plasma, pg/ml)** |  | **VEGF-A (serum, pg/ml)** |  | **bFGF (serum, pg/ml)** |  |
| --- | --- | --- | --- | --- | --- | --- | --- | --- |
| **Overall response** | **yes** | **no** | **yes** | **no** | **yes** | **no** | **yes** | **no** |
| **Mean** | **30.6** | **14.1** | **72.2** | **114.1** | **342.0** | **391.3** | **7.1** | **7.1** |
| **SEM^a^** | **23.9** | **3.0** | **56.7** | **29.5** | **121.9** | **66.4** | **2.6** | **0.9** |
| **Median^*^** | **4.8** | **7.4** | **14.0** | **74.0** | **255.5** | **381.0** | **7.2** | **8.2** |
| **Minimum** | **0.9** | **1.1** | **0** | **0** | **66.0** | **18.0** | **0** | **0** |
| **Maximum** | **149.0** | **49.2** | **353.0** | **615.0** | **901.0** | **1110.0** | **15.4** | **11.8** |
| **Number of samples** | **6** | **22** | **6** | **23** | **6** | **22** | **6** | **22** |

a: Standard error of mean (SEM); p=0.49 (HSP27), p=0.28 (VEGFA, plasma), p=0.72 (VEGF-A, serum), p=0.89 (bFGF); *Mann-Whitney U test.
