# Supplementary material for: Expression of Heat Shock Protein 27 in Melanoma Metastases Is Associated with Overall Response to Bevacizumab Monotherapy: Analyses of Predictive Markers in a Clinical Phase II Study
Source: PLoS One. 2016 May 11;11(5):e0155242. doi: 10.1371/journal.pone.0155242 (PMC4864228; doi:10.1371/journal.pone.0155242)
Supplement: S12 Table — (DOCX) [file pone.0155242.s016.docx]

**S12 Table. Concentrations of HSP27, VEGF-A and bFGF in blood samples according to line of treatment**

a: Standard error of mean (SEM); p<0.0005 (HSP27), p=0.017 (VEGFA, plasma), p=0.072 (VEGF-A, serum), p=0.002 (bFGF);

*Mann-Whitney U test.

|  | **HSP27 (serum, ng/ml)** |  | **VEGF-A (plasma, pg/ml)** |  | **VEGF-A (serum, pg/ml)** |  | **bFGF (serum, pg/ml)** |  |
| --- | --- | --- | --- | --- | --- | --- | --- | --- |
| **Bevacizumab treatment** | **1^st^ line** | **2^nd^ line** | **1^st^ line** | **2^nd^ line** | **1^st^ line** | **2^nd^ line** | **1^st^ line** | **2^nd^ line** |
| **Mean** | **7.1** | **29.8** | **53.3** | **169.6** | **267.1** | **511.9** | **4.6** | **10.0** |
| **SEM^a^** | **3.1** | **10.5** | **18.3** | **48.5** | **49.2** | **100.3** | **1.2** | **0.7** |
| **Median^*^** | **2.5** | **22.4** | **20** | **118.0** | **189.0** | **415.0** | **6.7** | **9.5** |
| **Minimum** | **0.9** | **6.0** | **0** | **0** | **18.0** | **113.0** | **0** | **7.3** |
| **Maximum** | **49.2** | **149.0** | **214** | **615.0** | **547.0** | **1110.0** | **10.7** | **15.4** |
| **Number of samples** | **15** | **13** | **16** | **13** | **15** | **13** | **15** | **13** |
